# Patient-reported pain and discomfort associated with lingual versus labial fixed orthodontic appliances: a systematic review

**DOI:** 10.1186/s41687-026-01166-2

**Published:** 2026-08-12

**Authors:** Shakfi Rahman, Abhita Malhotra, Dhruv Ahuja, Puneet Batra, Ashish Kumar Singh, Ashith MV

**Affiliations:** 1 Manav Rachna Dental College, Manav Rachna International Institute of Research and Studies (MRIIRS), Faridabad, Haryana, India; 2 Department of Orthodontics and Dentofacial Orthopaedics, Manipal College of Dental Sciences Mangalore, Manipal Academy of Higher Education, Manipal, Karnataka 576104 India

**Keywords:** Orthodontic pain, Labial appliances, Lingual appliances, Patient-reported outcomes

## Abstract

**Purpose of review:**

To systematically compare pain outcomes associated with labial and lingual fixed orthodontic appliances in patients undergoing active orthodontic treatment. A systematic review was conducted following PRISMA guidelines. Randomized clinical trials, prospective clinical controlled trials and retrospective observational studies reporting patient-reported pain were included. Databases searched included PubMed, Scopus, Web of Science, Cochrane Library, and EBSCO. Pain intensity, localization, and functional impact were extracted, and methodological quality was assessed using the Cochrane Risk of Bias 2.0 tool and Newcastle–Ottawa Scale. Due to heterogeneity in pain instruments, timing, and appliance design, meta-analysis could not be performed, and evidence certainty was evaluated using GRADE.

**Recent findings:**

Thirteen studies involving 775 participants, of whom 554 underwent direct comparison between labial and lingual orthodontic treatment, were included. Pain peaked within 24–48 h post-appliance placement and declined within 7–14 days. Lingual appliances were associated with tongue pain in 94.4% of patients compared with 50.0% in the labial group, whereas lip pain (52.8% vs. 76.3%) and cheek pain (50.0% vs. 86.8%) were less frequent in the lingual group. The overall certainty of evidence ranged from low to moderate according to GRADE assessment.

**Summary:**

All fixed orthodontic appliances cause transient early pain, with differences primarily in localization and functional impact. Lingual appliances pose greater early tongue discomfort and speech challenges, while labial appliances predominantly affect lips and cheeks. High-quality, standardized trials are needed to guide evidence-based appliance selection.

## Introduction

Pain is one of the most frequently reported adverse experiences associated with orthodontic treatment and may significantly influence patient compliance, treatment acceptance, and overall patient satisfaction. Miller et al. [1] reported that orthodontic discomfort typically begins within a few hours following appliance activation, reaches peak intensity at approximately 24 h, and gradually subsides thereafter, usually becoming minimal within 7–14 days. This pain is primarily attributed to the inflammatory response within the periodontal ligament following the application of orthodontic forces, resulting in localized pressure and sensitivity [2]. 

Fixed orthodontic appliances are commonly used to correct malocclusions and improve dental aesthetics and function. Among these, labial and lingual fixed orthodontic appliances are widely employed in contemporary orthodontic practice [3]. Labial appliances are bonded to the buccal surfaces of the teeth and represent the conventional orthodontic treatment modality. However, increasing demand for aesthetic treatment options has led to the development and growing popularity of lingual orthodontic appliances, which are bonded to the lingual surfaces of the teeth and are therefore less visible during treatment [4]. Despite the aesthetic advantages associated with lingual orthodontic appliances, their placement on the lingual surfaces of the teeth may result in specific functional challenges [5–7]. 

Previous clinical investigations have attempted to evaluate differences in patient-reported pain and discomfort associated with lingual and labial orthodontic appliances. Khattab et al. [2] reported that patients treated with lingual appliances experienced greater speech difficulties and chewing discomfort during the early stages of treatment. Similarly, Yuan et al. [3] observed comparable overall pain levels between lingual and labial appliances but noted differences in the localization of discomfort, with lingual appliances producing greater tongue-related irritation and labial appliances resulting in increased lip and cheek discomfort. Shalish et al. [4] also reported a longer adaptation period for patients undergoing lingual orthodontic treatment.

A previous systematic review [8] has evaluated the adverse effects associated with lingual orthodontic treatment. For instance, Ata-Ali et al. [8] conducted a systematic review and meta-analysis comparing lingual and labial orthodontic techniques and concluded that lingual appliances were associated with greater oral discomfort, speech difficulties, and eating impairment. However, the authors also emphasized that the available evidence was limited by methodological heterogeneity and variations in outcome assessment methods.

In recent years, several clinical studies evaluating patient-reported outcomes associated with lingual orthodontic appliances have been published, including investigations focusing on modern customized lingual appliance systems and their impact on patient comfort and adaptation [2–4, 6, 8–17]. These studies provide additional evidence regarding patient-reported pain outcomes and may contribute to a more comprehensive understanding of differences between lingual and labial orthodontic appliances.

Given the continuing advancements in orthodontic appliance design and the increasing emphasis on patient-reported outcomes in clinical research, an comprehensive synthesis of the available evidence focusing specifically on pain and discomfort associated with lingual and labial orthodontic appliances is warranted. Therefore, the present systematic review aimed to systematically evaluate and compare patient-reported pain and discomfort associated with lingual and labial fixed orthodontic appliances in patients undergoing active orthodontic treatment.

## Materials and methods

### Study design and protocol registration

This systematic review was conducted in accordance with the Preferred Reporting Items for Systematic Reviews and Meta-Analyses (PRISMA) guidelines [18]. The review protocol was prospectively registered in the International Prospective Register of Systematic Reviews (PROSPERO) under the registration number CRD42025639221. The research question was formulated using the Population, Intervention, Comparison, and Outcome (PICO) framework to guide the study selection and data extraction process. Following PROSPERO registration, minor methodological enhancements were made to improve the comprehensiveness and transparency of the review. These included the addition of the Cochrane Library as an electronic database, explicit specification of eligible study designs (randomized controlled trials, prospective clinical studies, and retrospective observational studies), and expansion of the secondary patient-reported outcomes to include tongue soreness, lip and cheek irritation, chewing discomfort, speech difficulty, analgesic consumption, functional impairment, oral health-related quality of life (OHRQoL), and general quality of life. Google Scholar was initially considered as a supplementary search source in the protocol; however, it was not included in the final electronic database search because of limited reproducibility and extensive duplicate retrieval. Instead, manual screening of the reference lists of included studies and relevant systematic reviews was undertaken to identify additional eligible studies. These methodological refinements did not alter the primary objective, research question, or eligibility framework of the review.

### Eligibility criteria

Studies were considered eligible for inclusion if they evaluated patient-reported pain or discomfort associated with lingual and labial fixed orthodontic appliances in patients undergoing active orthodontic treatment. Randomized controlled trials, prospective clinical studies, and retrospective observational studies were considered eligible, provided that they directly compared lingual and labial orthodontic appliances and reported pain-related outcomes using validated assessment tools.

Studies were excluded if they were in vitro studies, case reports, narrative reviews, systematic reviews, or meta-analyses. Studies that did not include a direct comparison between lingual and labial orthodontic appliances or those that did not report patient-reported pain outcomes were also excluded. Google Scholar was not used as a primary electronic database because of limited search reproducibility and extensive duplicate retrieval. Additionally, studies involving patients with craniofacial syndromes or systemic conditions that could influence pain perception were excluded to minimize potential confounding factors.

### Information sources and search strategy

A comprehensive electronic literature search was performed to identify relevant studies evaluating patient-reported pain outcomes associated with lingual and labial orthodontic appliances. The search was conducted in the following electronic databases: PubMed/MEDLINE, Scopus, Web of Science, the Cochrane Library, and EBSCOhost (Dentistry & Oral Sciences Source). This publication period was consistent with the eligibility criteria specified in the registered protocol.

The search strategy combined Medical Subject Headings (MeSH) and free-text keywords related to orthodontics, appliance type, and pain outcomes. Boolean operators such as **AND** and **OR** were used to combine the search terms. A representative search strategy used for each database is provided in Appendix 1.

In addition to the electronic database search, the reference lists of relevant articles and previously published systematic reviews were manually screened to identify additional eligible studies that may not have been captured through the electronic search process.

### Study selection

All identified records were imported into reference management software, and duplicate articles were removed prior to the screening process. Two independent reviewers screened the titles and abstracts of all retrieved studies to determine their relevance according to the predefined eligibility criteria. Studies that appeared to meet the inclusion criteria or those for which eligibility could not be determined from the title and abstract were selected for full-text evaluation.

Full-text articles were then independently assessed by the reviewers to determine final eligibility. Any disagreements regarding study selection were resolved through discussion between the reviewers, and when necessary, consultation with a third reviewer was undertaken to reach consensus. Inter-reviewer agreement (e.g., Cohen’s κ) was not formally calculated.

### Data extraction

Data extraction was independently performed by two reviewers using a standardized data extraction form developed for this review. Information extracted from each included study included the author and year of publication, study design, sample size, participant characteristics, type of orthodontic appliance used, pain assessment instruments, timing of pain evaluation, and the main outcomes related to patient-reported pain and discomfort.

The extracted data were carefully cross-checked by the reviewers to ensure accuracy and consistency. Any discrepancies identified during the data extraction process were resolved through discussion and consensus.

### Risk of bias assessment

The methodological quality of the included studies was assessed using appropriate tools based on the study design. Randomized controlled trials were evaluated using the Cochrane Risk of Bias 2.0 tool, which assesses potential bias across several domains including randomization process, deviations from intended interventions, missing outcome data, outcome measurement, and selective reporting [19]. 

Observational studies were evaluated using the Newcastle–Ottawa Scale (NOS), which assesses study quality based on the selection of study groups, comparability of groups, and assessment of outcomes [20]. Each study was independently evaluated by two reviewers, and any disagreements were resolved through discussion. Inter-reviewer agreement (e.g., Cohen’s κ) was not formally calculated.

### Data synthesis

Due to heterogeneity among the included studies with respect to study design, pain assessment instruments, timing of pain evaluation, and orthodontic appliance systems used, a quantitative meta-analysis was considered inappropriate. Therefore, a qualitative synthesis of the included studies was performed to summarize and interpret the available evidence.

The certainty of evidence for the outcomes was assessed using the Grading of Recommendations Assessment, Development and Evaluation (GRADE) framework, which evaluates the overall strength of evidence based on factors such as study limitations, consistency of results, precision of estimates, and risk of bias [21]. 

## Results

### Study selection

The electronic database search identified a total of 833 records across the selected databases. After removal of duplicate articles, 511 records remained for title and abstract screening. During the initial screening process, studies that were clearly unrelated to the research question or did not meet the inclusion criteria were excluded. Following this step, 23 full-text articles were retrieved and assessed for eligibility.

**Fig. 1:**
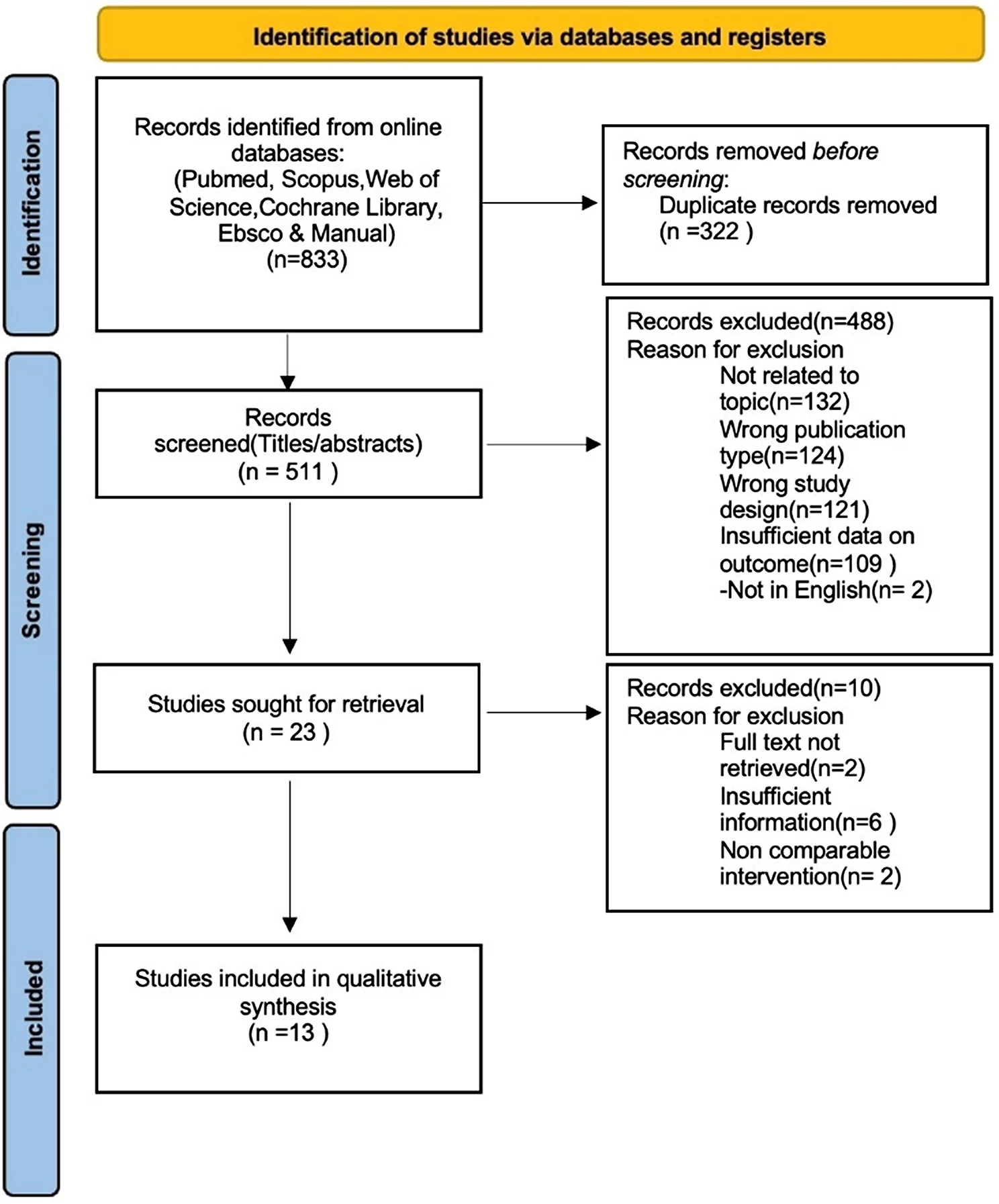
PRISMA flow diagram

After detailed evaluation of the full-text articles according to the predefined inclusion and exclusion criteria, 13 studies were found to meet the eligibility criteria and were included in the final qualitative synthesis [2–4, 6, 8–17]. Where multiple publications from the same research group were identified, the studies were carefully examined for potential overlap in participant cohorts. If publications appeared to report data from the same or substantially overlapping study population, participant numbers were counted only once to avoid duplication, while unique outcomes reported in separate publications were retained for the qualitative synthesis. The reasons for exclusion of the remaining studies included lack of direct comparison between lingual and labial orthodontic appliances, absence of patient-reported pain outcomes, or study designs that did not meet the eligibility criteria. The process of study identification, screening, eligibility assessment, and final inclusion is summarized in the PRISMA flow diagram (Fig. 1).

### Study characteristics

The thirteen included studies consisted of randomized controlled trials, prospective clinical studies, and retrospective observational investigations evaluating patient-reported pain and discomfort associated with lingual and labial orthodontic appliances [2–4, 6, 8–17]. In total, the included studies involved 775 participants undergoing orthodontic treatment, of which 554 participants were included in labial and lingual groups (Table 1).

**Table 1:** Characteristics of included studies comparing lingual and labial orthodontic appliances

Author and Year	Study Design & Sample	Age	Intervention / Brackets Used	Methodology	Time of Evaluation	Objective	Outcome
Caniklioglu C et al., 2005 [6]	Prospective controlled clinical trial (CCT) *n* = 60 (LI = 30, LA = 30)	LI: Mean 17 years 7 months; LA: Mean 18 years 3 months	Lingual – Ormco 7th generation (Glendora, California); Labial – Roth brackets (Ormco)	Questionnaire-measures pain intensity, duration severity	After 3 months	To determine differences in discomfort between lingual and labial orthodontic appliances	Both groups experienced soft tissue discomfort initially, which gradually subsided within one month. Tongue soreness was more common in the lingual group (*P* < 0.001), while lip (*P* < 0.05), and cheek (*P* < 0.001), discomfort was more frequent in the labial group. No significant differences between LA and LI group in the intraoral discomfort
Wu AK et al., 2010 [9] and Wu A et al., 2011 [10]	Prospective controlled clinical trial *n* = 60 (LI = 30, LA = 30)	Labial: Mean 20.33 years; Lingual: Mean 21.63 years	Lingual – Incognito (Bad Essen, Germany); Labial – Mini-Diamond (Ormco, California)	VAS (100 mm)-measures pain intensity	T1: 1 week; T2: 1 month; T3: 3 months	To compare pain experiences among Chinese adult patients treated with labial and lingual orthodontic appliances	No significant differences in pain scores between the two groups (*P* > 0.05). Lingual appliances caused more tongue pain (*P* < 0.001), whereas labial appliances caused more lip (*P* < 0.001), and cheek (*P* < 0.001), pain.
Shalish M et al., 2012 [4]	Prospective controlled clinical trial *n* = 68 (LI = 19, LA = 28, Aligners = 21)	Adults (18–60 years)	Lingual – Incognito (Bad Essen, Germany); Labial – Straight wire appliance (GAC and Ormco); Aligners – Invisalign	HRQoL Questionnaire-measures pain perception and dysfunction	1st week and 14th day	To evaluate perceived recovery in adults treated with labial, lingual, and Invisalign orthodontic appliances	Lingual appliances were associated with more severe pain, greater analgesic consumption, and longer recovery compared with labial appliances.(*P* = 0.003),
Khattab TZ et al., 2013 [2]	Randomized controlled trial *n* = 34 (LI = 17, LA = 17)	15–30 years	Labial – Roth brackets (Ormco); Lingual – StealthH (American Orthodontics)	Questionnaire- measures oral impairment	Before bonding (T1), immediately after bonding (T2), 1 month, and 3 months after (T3)	To compare levels of oral impairment between lingual and labial appliances	Both appliances caused soft-tissue irritation; however, irritation was significantly greater with lingual appliances initially but decreased over three months. LI at T1 and T2 statistically significant (*P* < 0.001), T3 not significant; LA at T1 statistically significant (*P* < 0.009), T2 not significant, T3 NA
Antonio-Zancajo L et al., 2020 [11] and Antonio-Zancajo L et al., 2021 [13]	Prospective controlled clinical trial *n* = 120 (LI = 30, LA = 30, LF = 30, Aligners = 30)	18–40 years	Labial – Victory Series (3 M); Low-friction – Synergy (Rocky Mountain Orthodontics); Lingual – STB (Ormco); Aligners – Invisalign	McGill Pain Questionnaire; measures pain and OHIP-14- measures oral health-related quality of life.	4, 8, and 24 h; days 2–7	To compare pain and its relationship with oral quality of life among different orthodontic appliances	Peak pain occurred within the first 24 h and decreased during the first week. A statistically significant difference was seen between the two groups (*P* < 0.001). VAS score for LA (3.8 ± 2.3) was more than LI (1.7 ± 2.5)
AlSeraidi M et al., 2021 [12]	Prospective controlled clinical trial *n* = 117 (LI = 37, LA = 41, Aligners = 39)	> 18 years	Labial – Mini Clippy (TOMY, Japan); Lingual – Incognito (3 M Unitek); Aligners – Flash Orthodontics	WHOQoL-BREF Questionnaire measures quality of life.	6 weeks	To evaluate overall quality of life across physical, psychological, social, and environmental domains	No statistically significant difference (*P* > 0.05) was seen between LA (22.7 ± 2) and LI (22 ± 1.9) groups in the physical aspect of QoL.
Zamora-Martínez N et al., 2021 [14]	Prospective controlled clinical trial *n* = 120 (LA = 30 LI = 30 CB = 30 Aligners = 30)	18–68 years	Labial metal brackets, ceramic brackets, lingual brackets, and aligners	OHIP questionnaire measures quality of life	Before treatment, 6 months, end of treatment	To analyze changes in oral health-related quality of life during orthodontic treatment	Lingual showed significantly better OHRQoL at 6 months (*P* = 0.018); no difference at baseline or treatment completion.
Kara-Boulad JM et al., 2022 [16]	Randomized controlled trial *n* = 38 (LI = 19, LA = 19)	18–25 years	Labial – Mini Master (American Orthodontics); Lingual – DTC Orthodontics	OHIP-14 Questionnaire measures quality of life.	Baseline(T0), 1 week(T1), 1 month(T2), 3 months (T3), 6 months(T4), end of treatment (T5)	To compare OHRQoL between lingual and labial appliances	Lingual appliances caused significantly greater physical pain than labial appliances throughout treatment (1 week: 5.95 vs. 4.16, *P* < 0.001; 1 month: 3.68 vs. 2.37, *P* < 0.001; 3 months: 2.21 vs. 1.21, *P* < 0.001; 6 months: 0.89 vs. 0.53, *P* = 0.025; end of treatment: 0.68 vs. 0.32, *P* = 0.023).
Özkalaycı N et al., 2023 [15]	Retrospective study *n* = 30 (LI = 15, LA = 15)	Not reported	Lingual – Discovery Delight (Dentaurum); Labial – Equilibrium 2 (Dentaurum)	Questionnaire -measures pain intensity and the nature (quality) of pain.	Days 1(T0), 7(T1), and 14(T2)	To compare effects of lingual and labial appliances on patient comfort	At T0, there was no significant difference between the groups in terms of pain level (*p* > 0.05). The tongue trauma in LI group was more than LA group (*p* < 0.05). Significant decreases were seen between T0-T1, T0-T2 and T1-T2 periods in terms of pain level (*p* < 0.05).
Yuan L et al., 2024 [3]	Prospective controlled clinical trial *n* = 115 (LI = 36, LA = 38, MXD = 41)	> 18 years	Customized lingual, labial self-ligating, and mixed appliances	VAS (10 cm scale) for comfort assessment	0, 2, 6 h; days 1, 3, 7, 14, 21	To compare discomfort and functional impairment with different appliance systems	LI group showed significantly lower lip pain (52.75% vs. 76.32%; *P* = 0.0044) and cheek pain (50.00% vs. 86.84%; *P* = 0.0003), but significantly higher tongue pain (94.44% vs. 50.00%; *P* < 0.0001) compared with LA group.
Alzainal MH et al., 2024[17]	Randomized controlled trial *n* = 13 (LI = 6, LA = 7)	10–18 years	Labial – Victory Series (3 M); Lingual – BRIUS	VAS (100 mm) Questionnaire- measures discomfort	Daily for 7 days	To evaluate discomfort after appliance placement	LI group showed significantly greater tongue discomfort (*p* < 0.05, days 2–7), whereas LA showed significantly greater cheek discomfort (day 1: *p* = 0.032; day 2: *p* = 0.023). No significant differences were observed in tooth, gingival, or lip discomfort.

Pain and discomfort were assessed using various validated instruments across the included studies. The Visual Analog Scale (VAS) was the most commonly used method for evaluating pain intensity [3, 9, 10, 17]. Other studies utilized standardized questionnaires such as the McGill Pain Questionnaire, Oral Health Impact Profile (OHIP-14), and WHOQOL-BREF to evaluate both pain and oral health–related quality of life [2, 4, 6], [11–16]. The timing of pain assessment varied among studies; however, most investigations evaluated pain intensity within the first few hours or days following appliance placement and at subsequent follow-up intervals extending up to several weeks or months [3, 11, 13, 17] (Table 1).

In addition to pain intensity, several studies also evaluated functional outcomes including speech difficulties, chewing discomfort, and oral health–related quality of life associated with the use of different orthodontic appliance systems [4, 12, 14, 16]. 

### Risk of bias assessment

The randomized controlled trials generally demonstrated low risk of bias for the randomization process, missing outcome data, and measurement of outcomes. Some concerns were identified regarding deviations from intended interventions and selection of the reported results, resulting in an overall judgment of “some concerns” for all three randomized controlled trials (Table 2) [2, 16, 17]. In contrast, the observational studies assessed using the Newcastle–Ottawa Scale generally showed good methodological quality, with concerns primarily related to participant selection, comparability of study groups (control of confounding), outcome assessment, and adequacy of follow-up (Table 3) [3, 4, 6, 9–15]. Prospective controlled clinical studies without true randomization were appraised using the Newcastle–Ottawa Scale because they lacked true randomization and therefore were methodologically comparable to cohort studies, making the Newcastle–Ottawa Scale the most appropriate appraisal tool. Nevertheless, variations in study design, pain assessment instruments, and follow-up duration contributed to methodological heterogeneity among the included studies.

**Table 2:** Cochrane risk of bias assessment for randomized controlled trial

Study	Domain 1: Randomization Process	Domain 2: Deviations from Intended Interventions	Domain 3: Missing Outcome Data	Domain 4: Measurement of the Outcome	Domain 5: Selection of the Reported Result	Overall Risk of Bias
Khattab et al., 2013[2]	Low	Some concerns	Low	Low	Some concerns	Some concerns
Kara-Boulad et al., 2022[16]	Low	Some concerns	Low	Low	Low	Some concerns
Alzainal et al., 2024[17]	Low	Some concerns	Low	Low	Low	Some concerns

**Table 3:** Newcastle–ottawa quality assessment of cohort studies

Study	Representativeness of Exposed Cohort	Selection of Non-exposed Cohort	Ascertainment of Exposure	Outcome Not Present at Start	Comparability	Outcome Assessment	Adequate Follow-up	Total Score
Caniklioglu 2005 [6]	★	★	★	★	★★	★	★	7
Wu AK 2010 [9]	★	★	★	★	★★	★	★	7
Wu A 2011 [10]	★	★	★	★	★★	★	★	7
Shalish 2012 [4]	★	★	★	★	★	★	★	6
Antonio-Zancajo 2020 [11]	★	★	★	★	★★	★	★	7
Antonio-Zancajo 2021 [13]	★	★	★	★	★★	★	★	7
AlSeraidi 2021 [12]	★	★	★	★	★	★	★	6
Zamora-Martínez 2021 [14]	★	★	★	★	★★	★	★	7
Özkalaycı 2023 [15]	★	★	★	★	★★	★	★	7
Yuan 2024 [3]	★	★	★	★	★★	★	★	7

### Pain outcomes

Across the included studies, orthodontic appliance placement was consistently associated with an increase in pain and discomfort during the early phase of treatment. Pain intensity typically began within a few hours following appliance placement and reached its peak within the first 24–48 h, after which pain levels gradually declined as patients adapted to the orthodontic appliances [2, 3, 11]. 

Several studies reported that patients treated with lingual orthodontic appliances experienced greater tongue irritation, speech difficulties, and chewing discomfort during the initial stages of treatment [2, 4, 16, 17]. These findings were attributed to the lingual positioning of the brackets, which may interfere with tongue movement and speech articulation.

In contrast, patients treated with labial orthodontic appliances more frequently reported irritation of the lips and buccal mucosa, which is consistent with the placement of brackets on the buccal surfaces of the teeth [3, 6, 17]. Although the area of discomfort differed between appliance types, the available evidence did not show a consistent difference in overall pain intensity. Findings were heterogeneous across studies, with some reporting greater pain and discomfort associated with lingual appliances, others reporting lower pain scores or better patient-reported outcomes with lingual appliances, and several reporting no significant difference between the two appliance systems [4, 9–17].

### Functional and quality-of-life outcomes

In addition to pain intensity, several included studies evaluated functional limitations and oral health–related quality of life associated with orthodontic treatment [2, 4, 6, 11–16]. Patients undergoing lingual orthodontic treatment reported greater initial difficulties related to speech and tongue irritation, particularly during the early stages of treatment [2, 4, 16, 17]. However, many studies reported that these functional limitations gradually improved as patients adapted to the appliances [12, 13]. Conversely, labial orthodontic appliances were associated with increased irritation of the lips and cheeks but generally resulted in fewer speech-related difficulties [3, 6, 17]. Some investigations also suggested that the aesthetic advantage of lingual orthodontic appliances contributed to improved psychological satisfaction among patients despite the initial discomfort [11, 14]. 

### Evidence synthesis

Due to the heterogeneity among the included studies with respect to study design, pain assessment instruments, orthodontic appliance systems, and follow-up duration, a quantitative meta-analysis was not considered appropriate. Therefore, a qualitative synthesis of the available evidence was performed to summarize the findings of the included studies. The certainty of evidence ranged from low to moderate. Evidence for overall pain intensity and early pain was rated as moderate because of inconsistency and methodological limitations across studies. Evidence for pain localization, functional impairment, and OHRQoL was downgraded to low certainty owing to study design limitations, heterogeneity in outcome assessment, imprecision arising from small sample sizes, and inconsistency across study findings [21]. (Table 4) Overall pain intensity and early pain started at high certainty because evidence was primarily derived from randomized and prospective comparative studies, but were downgraded one level for risk of bias and inconsistency. Pain localization, functional impairment, and OHRQoL were additionally downgraded for imprecision resulting in low certainty.

**Table 4:** GRADE summary of evidence

Outcome	No. of Studies (Participants)	Study Design	Risk of Bias	Inconsistency	Indirectness	Imprecision	Publication Bias	Overall Certainty
Overall pain intensity	13 studies (775 patients)	3 RCTs, 9 pCCTs, 1 retrospective study	Moderate	Moderate	Low	Moderate	Low	**MODERATE **⊕⊕⊕◯
Early pain (24–72 h)	10 studies (631 patients)	RCTs and pCCTs	Moderate	Moderate	Low	Moderate	Low	**MODERATE **⊕⊕⊕◯
Pain localization	9 studies (650 patients)	Prospective studies	Moderate	Low	Low	Moderate	Low	**LOW **⊕⊕◯◯
Functional impairment	6 studies (420 patients)	Prospective studies + 1 RCT	Moderate	Moderate	Low	Moderate	Low	**LOW **⊕⊕◯◯
OHRQoL	5 studies (390 patients)	Prospective clinical studies	Moderate	Moderate	Low	Moderate	Low	**LOW **⊕⊕◯◯

Downgraded for: a = risk of bias, b = inconsistency, c = imprecision

## Discussion

The present systematic review aimed to compare the pain outcomes associated with lingual and labial fixed orthodontic appliances in patients undergoing active orthodontic treatment. Pain and discomfort are among the most frequently reported adverse effects of orthodontic therapy and can significantly influence patient compliance and treatment experience [1]. Understanding the differences in patient-reported pain between orthodontic appliance systems is therefore clinically relevant for both orthodontists and patients when selecting an appropriate treatment modality.

The findings of the present review suggest that both lingual and labial orthodontic appliances are associated with transient pain and discomfort during the early stages of treatment, particularly within the first 24–48 h following appliance placement, after which pain levels gradually decline as patients adapt to the appliances [2, 3, 10, 12]. These findings are consistent with the biological response to orthodontic force application, which involves inflammatory processes within the periodontal ligament that lead to temporary discomfort.

An important finding of the present review is that the localization and functional impact of discomfort differed consistently according to the appliance type. Patients treated with lingual orthodontic appliances experienced greater initial tongue irritation and speech-related difficulties, primarily due to the lingual positioning of brackets, which may interfere with normal tongue movement and articulation [2, 6, 17]. Conversely, labial orthodontic appliances were more frequently associated with irritation of the lips and buccal mucosa, reflecting the location of brackets on the buccal surfaces of teeth [3, 10]. These findings indicate that although both appliance systems produce treatment-related discomfort, the affected anatomical sites and functional limitations differ according to the appliance design.

In contrast, the available evidence suggests no consistent difference in overall pain intensity between lingual and labial orthodontic appliances. However, this finding should be interpreted cautiously because the included studies used different pain assessment instruments, evaluated patients at different follow-up intervals, investigated different appliance systems, and varied in study design. Consequently, direct comparison of overall pain intensity across studies is limited, and the certainty of this conclusion remains moderate [2–4, 10, 12]. 

The GRADE assessment indicated that the certainty of evidence for most outcomes ranged from low to moderate. This reflects methodological limitations of the included studies, including heterogeneity in pain assessment instruments, variability in follow-up periods, differences in orthodontic appliance systems, and inconsistency across study findings. Therefore, although the available evidence provides useful clinical insights, the findings should be interpreted with caution and should not be considered definitive.

The findings of the present review are generally consistent with previous systematic reviews that have investigated adverse effects associated with different orthodontic appliance systems. For instance, the systematic review and meta-analysis by Ata-Ali et al. reported no significant differences in overall pain intensity between lingual and labial orthodontic appliances, although lingual appliances were associated with greater speech disturbances and tongue discomfort [8]. Similarly, Long et al. reported that both appliance systems produced comparable levels of treatment-related discomfort, whereas functional limitations varied with appliance design [3]. 

However, the present review differs from previous reviews in several important aspects. First, more recently published studies evaluating patient-reported pain outcomes and oral health–related quality of life were included in the analysis [2, 4, 6, 11–16]. These studies provided additional insights into the patient experience during orthodontic treatment, particularly regarding functional adaptation and quality-of-life outcomes associated with lingual orthodontic appliances. Second, the present review incorporated a GRADE-based evaluation of the certainty of evidence, allowing a more structured assessment of the strength of the available evidence.

Another important consideration is that pain perception during orthodontic treatment is inherently subjective and multifactorial, and may be influenced by several factors including individual pain thresholds, psychological status, cultural background, and the quality of patient education and support provided during treatment [7]. Effective communication between the clinician and patient regarding expected discomfort during orthodontic therapy may therefore play an important role in improving patient acceptance and compliance.

### Limitations and future scope

Although the present systematic review followed a structured methodology and included multiple databases, certain limitations should be acknowledged. The included studies demonstrated considerable heterogeneity with respect to study design, pain assessment instruments, and follow-up periods. In addition, some studies included relatively small sample sizes or observational study designs, which may introduce potential bias. Due to these methodological differences, a quantitative meta-analysis was not considered appropriate.

In addition, although publications from the same research groups (e.g., Wu et al., 2010/2011 and Antonio-Zancajo et al., 2020/2021), were carefully assessed for potential overlap in study populations and duplicate participant counting was avoided, the possibility of residual overlap cannot be completely excluded.

Furthermore, the limited number of high-quality randomized controlled trials and the overall low to moderate certainty of evidence highlight the need for further well-designed clinical trials using standardized pain assessment tools, validated patient-reported outcome measures and longer follow-up periods. Future studies should also consider evaluating additional patient-centered outcomes such as treatment satisfaction, speech adaptation, and long-term oral health–related quality of life.

Despite these limitations, the present review provides a comprehensive synthesis of the available evidence regarding pain outcomes associated with lingual and labial orthodontic appliances and may help clinicians better inform patients about the expected treatment experience.

## Conclusion

Within the limitations of this systematic review, both lingual and labial fixed orthodontic appliances are associated with temporary pain and discomfort during the early stages of orthodontic treatment. While lingual appliances may result in greater initial tongue irritation and speech-related difficulties, labial appliances are more commonly associated with irritation of the lips and buccal mucosa. Overall, the available evidence suggests no consistent difference in overall pain intensity between lingual and labial appliances, but certainty is limited by heterogeneity in outcome measures, follow-up timing, appliance systems and study design. The nature and localization of discomfort may differ depending on the appliance system. Further high-quality randomized controlled trials with standardized outcome measures are required to provide more definitive evidence regarding patient-reported pain and functional outcomes associated with different orthodontic appliance systems.

## Appendix 1: Search strategy 

**Table:** 

Database	Search terms	Results
PubMed	(“orthodontic appliances” OR “orthodontic treatment”) AND (labial OR ‘’labial orthodontics’’ OR ‘’labial appliance’’ OR ‘’labial bracket’’ OR conventional OR “vestibular appliance”) AND (lingual OR ‘’lingual orthodontics’’ OR ‘’lingual bracket’’) AND (Pain OR discomfort OR “Quality of life” OR “oral impairment” OR effects OR “oral impact”)	457
EBSCO	(“orthodontic appliances” OR “orthodontic treatment”) AND (labial OR ‘’labial orthodontics’’ OR ‘’labial appliance’’ OR ‘’labial bracket’’ OR conventional OR “vestibular appliance”) AND (lingual OR ‘’lingual orthodontics’’ OR ‘’lingual bracket’’) AND (Pain OR discomfort OR “Quality of life” OR “oral impairment” OR effects OR “oral impact”)	91
Cochrane Library	(“orthodontic appliances” OR “orthodontic treatment”) AND (labial OR ‘’labial orthodontics’’ OR ‘’labial appliance’’ OR ‘’labial bracket’’ OR conventional OR “vestibular appliance”) AND (lingual OR ‘’lingual orthodontics’’ OR ‘’lingual bracket’’) AND (Pain OR discomfort OR “Quality of life” OR “oral impairment” OR effects OR “oral impact”)	52
Scopus	((“orthodontic appliances” OR “orthodontic treatment”) AND (labial OR conventional OR vestibular) AND (lingual) AND (pain OR discomfort OR “quality of life” OR OHRQoL OR impairment))	123
Web of science	((“orthodontic appliances” OR “orthodontic treatment”) AND (labial OR conventional OR vestibular) AND (lingual) AND (pain OR discomfort OR “quality of life” OR OHRQoL OR impairment))	110

## Data Availability

Data is provided within the manuscript or supplementary information files.

## Declarations

### Generative AI in scientific writing

During the preparation of this manuscript, the authors have not used AI for scientific writing. The authors critically reviewed, revised, and approved the final content and accept full responsibility for the accuracy and integrity of the manuscript.
